# Appropriateness of Empiric Initiation of Meropenem in the Intensive Care Unit as Determined by Internal Medicine Residents

**DOI:** 10.1017/ash.2024.410

**Published:** 2024-10-24

**Authors:** Ufaq Ishtiaq, Katherine Acosta, Chika Akabusi, Kelcey Noble, Nili Gujadhur, Valerie Cluzet

**Affiliations:** 1 Department of Medicine, Nuvance Health at Vassar Brothers Medical Center, Poughkeepsie, NY, USA; 2 Division of Pulmonary Disease and Critical Care Medicine, Department of Medicine, University of Vermont Medical Center, Burlington, VT, USA; 3 Department of Medicine, Presbyterian Hospital, Albuquerque, NM, USA; 4 Department of Pharmacy, Nuvance Health at Vassar Brothers Medical Center, Poughkeepsie, NY, USA; 5 Division of Infectious Diseases, Department of Medicine, Nuvance Health at Vassar Brothers Medical Center, Poughkeepsie, NY, USA

## Abstract

**Objective:**

To evaluate the appropriateness of empiric initiation of meropenem in the intensive care unit (ICU) and to determine the agreement between internal medicine (IM) residents and infectious diseases (ID) physicians/pharmacists on appropriateness.

**Design:**

Retrospective observational study.

**Setting:**

ICU in a tertiary care community teaching hospital.

**Participants:**

Adult patients admitted to the ICU and started empirically on meropenem between April 1 and October 31, 2021.

**Methods:**

Meropenem usage was categorized as appropriate or inappropriate according to criteria developed from previously published indications and modified by ID physicians/pharmacists to reflect local practices. Two investigators (an IM resident and either an ID physician or pharmacist) assessed the appropriateness, with a second ID physician resolving any disagreements. Inter-rater reliability was measured using the kappa statistic.

**Results:**

Ninety-seven participants were enrolled, with a mean age of 68 (SD, 17.0) years. Pneumonia was the most common infection (30.9%). Among the participants, 92.8% received an ID consultation, with 55.6% of these occurring before meropenem initiation. IM residents deemed 56.7% of meropenem administrations appropriate, whereas an ID physician/pharmacist deemed only 48.5% appropriate, agreeing on 79.4% of cases (kappa statistic 0.59, *P* <.001). After a third reviewer’s assessment was included, agreement between the resident and at least one of the two reviewers reached 90.7% (kappa 0.81, *P* <.001).

**Conclusions:**

Approximately half of empiric meropenem started in the ICU was deemed inappropriate using institution-specific criteria. There was good agreement between IM residents and ID physicians/pharmacists on meropenem appropriateness. IM residents could contribute to antimicrobial stewardship efforts, like prospective audit and feedback, using standardized criteria for appropriateness.

## Introduction

Managing severe infections in the intensive care unit (ICU) and selecting appropriate empiric antibiotics are challenging. When infection is suspected, prompt initiation of antibiotics, ideally in the first hour, is required to improve outcomes.^1^ The selection of empiric antibiotic therapy should be based on the severity of illness, source of infection, patient risk factors (eg, immunosuppression, recent antibiotic use), medication allergies, local susceptibility patterns, and susceptibility of prior organisms identified.^2^ Initiation of inappropriate empiric antimicrobials is associated with increased morbidity and mortality rate, healthcare costs, and the development of antimicrobial resistance (AMR).^3^ The Centers for Disease Control and Prevention estimated that the cost of AMR is $55 billion every year in the United States: $20 billion for direct healthcare costs and about $35 billion for loss of productivity.^4^

Meropenem is a broad-spectrum antibiotic of the carbapenem family used for severe infections caused by multidrug-resistant organisms (MDRO). Meropenem is a useful medication to use empirically when there is a suspected MDRO infection or definitively for confirmed MDRO infections due to its broad-spectrum activity and low toxicity profile.^5^ The misuse of meropenem is associated with the development of carbapenem-resistant Enterobacterales, which are estimated to cause 19,000–49,000 infections annually in the United States.^6^ The prevention and control of MDRO have become a public health priority.^7,8^

The literature reveals that inappropriate use of meropenem is a prevalent problem. Studies evaluating meropenem use in hospitals have reported that 21%–46.5% of meropenem prescriptions are inappropriate.^9–12^ The studies evaluating the appropriateness of meropenem use have been performed by pharmacists or infectious diseases (ID) specialists.^13,14^ No studies reporting internal medicine (IM) resident-led assessment of the appropriateness of meropenem use were found. The aim of this study, therefore, was to assess the appropriateness of empiric initiation of meropenem in the ICU at a tertiary care community teaching hospital but also to determine the agreement between IM residents and ID attendings/pharmacists on assessment of appropriateness.

## Methods

### Study design, participants, and data collection

This was a retrospective observational study that was conducted by reviewing the electronic medical records of adult patients (≥18 years old) who were admitted to the ICU of a tertiary care community teaching hospital and received at least 1 dose of meropenem as empiric therapy between April 1^st^ and October 31^st^, 2021. Patients with available culture data for active infection and those started on meropenem prior to transfer to the ICU were excluded.

At our institution, many broad-spectrum and high-toxicity antimicrobials can be ordered by front-line providers, but the order includes a mandatory ID consult within 24 hours, and the ID physician must then approve the agent to continue. At the time of the study, this restriction on meropenem did not apply to critical care physicians but did apply to other specialties (cardiology, surgery, etc.). Daily prospective audit and feedback (PAF) was started in July 2021 but only for new starts of the unrestricted agents of piperacillin-tazobactam and ceftriaxone. Additionally, there were urinary tract infection (UTI) treatment guidelines that recommended empiric meropenem for patients with a history of extended-spectrum beta-lactamase (ESBL)-producing organisms. There were no specific guidelines relating to empiric carbapenem indications. Per the contemporary antibiogram, approximately 10% of systemic and urinary isolates of *Escherichia coli* and *Klebsiella pneumoniae* were ESBL-producing organisms.

Meropenem use was classified as appropriate or inappropriate based on criteria developed from previously published indications and risk factors modified by ID specialists and the ID pharmacist involved in the study to reflect local practices.^15–19^ Appropriate empiric initiation of meropenem was defined using the criteria listed in Table 1. Severe infection was defined as sepsis (life-threatening infection with at least one sign of organ dysfunction, elevated creatinine, transaminitis, new or worsening change in mental status, or use of supplemental oxygen) or septic shock (sepsis refractory to guideline-directed intravenous fluid hydration and requiring vasopressor support) or severe neutropenic fever (absolute neutrophil count <500/mL and temperature ≥ 101°F or 38.3°C). Risk factors for ESBL include long-term care facility resident, intravenous antibiotic use in the last 3 months, hemodialysis or presence of an intravascular catheter, percutaneous feeding tube, or indwelling urinary catheter.^20–27^ Criteria selection was based on clinical judgment and a holistic review of the medical record.

**Table 1.** Criteria for empiric use of meropenem^15–19^

1. Severe infection^*^ and history of infection or colonization with extended-spectrum beta-lactamase (ESBL) producing organism or multidrug-resistant *Pseudomonas aeruginosa* infection in the past 6 mo
2. Severe infection^*^ and at least 1 risk factor^ for ESBL-producing organisms in the past 6 mo
3. Severe infection^*^ and severe penicillin allergy (anaphylaxis)
4. Persistent fever or hemodynamic instability in a patient who is already receiving broad-spectrum β-lactam (agents with anti-pseudomonal coverage that are not carbapenems) for at least 48 h
5. Nosocomial bacterial meningitis

* Sepsis (life-threatening infection with at least one sign of organ dysfunction, elevated creatinine, transaminitis, new or worsening change in mental status, or use of supplemental oxygen) or septic shock (sepsis refractory to intravenous fluid hydration and requiring vasopressor support) or severe neutropenic fever (absolute neutrophil count <500/mL and temperature ≥ 101°F or 38.3°C). ^^^Long-term care facility resident, antibiotic use in the last 3 months, hemodialysis or presence of an intravascular catheter, percutaneous feeding tube, or indwelling urinary catheter.

The appropriateness of meropenem use was independently assessed by 2 investigators (1 IM resident and either a primary ID physician or ID pharmacist), both blinded to the others’ assessment until the review was complete. Conflicts in the assessment were resolved by a second ID physician review. A total of three residents participated, two PGY-3 and one PGY-2, at the time of the study. All three residents have pursued specialty training in pulmonary and critical care medicine.

Other variables collected included demographic data, duration of antibiotic administration, ICU and hospital length of stay (LOS), presence of ID consult, and in-hospital mortality.

### Statistical analysis

Descriptive statistics were performed using frequencies for categorical data and measures of central tendency and dispersion for continuous data, as appropriate. Outcomes were reported as frequencies and inter-rater reliability was measured using the kappa statistic. Characteristics of participants with meropenem orders deemed “appropriate” by either of the two ID physicians or ID pharmacist were compared to those with “inappropriate” prescriptions using the Mann–Whitney test, χ^2^ test, or Fisher’s exact test, as appropriate. Analysis was performed using Stata 18 (College Station, TX: StataCorp LLC). *P* values of <.05 were considered statistically significant.

## Results

During the study period, 130 patients on meropenem in the ICU were screened, but only 97 patients meeting the inclusion criteria were enrolled. Thirty-three patients, including duplicates and those meeting the exclusion criterion of meropenem initiation prior to ICU admission, were excluded. Table 2 shows the baseline characteristics of the participants. The mean age of participants was 68 (SD, 17 years) years, and approximately 76.3% were of white race. Pneumonia was the most prevalent infection upon admission (30.9%), and 79.4% of patients were admitted by the critical care team. The median ICU length of stay (LOS) was 5.3 days (interquartile range [IQR], 3–13), while hospital LOS was 14 days (IQR 8–28). In-hospital mortality was observed in 43.3% of patients. Of the 97 patients examined in the study, 90 (92.8%) received an ID consultation. Among those who received consultations, 50 patients (55.6%) had ID consultations prior to initiation of meropenem.

**Table 2.** Participant characteristics

Mean age	68 (SD, 17.0)
**Race**	
White	74 (76.3%)
Black or African American	12 (12.4%)
Other	11 (11.3%)
**Infection site**	
Pneumonia	30 (30.9%)
CNS	3 (3.1%)
Bloodstream	4 (4.1%)
SSTI	4 (4.1%)
Intra-abdominal	22 (22.9%)
UTI	24 (24.7%)
Febrile neutropenia	4 (4.1%)
Bone and joints	6 (6.2%)
**Admitting service**	
Surgery	10 (10.3%)
Trauma	1 (1.0%)
ICU	77 (79.4%)
Cardiology	9 (9.3%)
**In-hospital mortality**	
No	55 (56.7%)
Yes	42 (43.3%)
**Hospital stay and antibiotic duration (Days/Interquartile range [IQR])**	
Median LOS	14 (8, 28)
Median days of meropenem use	3 (1, 6)
Median LOS after stopping meropenem	3 (1, 9)
Median ICU LOS	5.3 (2.8, 12.6)
Median ICU LOS after stopping meropenem	4 (2,7)

Note. CNS, central nervous system; SSTI, skin and soft tissue infections; UTI, urinary tract infection, ICU, intensive care unit; LOS, length of stay.

Fifty patients were reviewed by a pharmacist, and 47 patients were reviewed by an ID physician. IM residents determined that 55 of the 97 patients (56.7%) were appropriately administered meropenem, whereas an ID physician/pharmacist determined that only 47 of the 97 patients (48.5%) received empiric meropenem appropriately. Of the 90 patients with ID consultation during ICU stay, 44 (51.1%) were deemed to have appropriate meropenem use. Agreement between the primary ID physician/pharmacist and IM resident was in 77 of the 97 (79.4%) patients (kappa 0.59, *P* <.001). Among the 20 disagreements between IM residents and ID physician/pharmacist, 7 disagreements (35%) were with the pharmacist, and 13 disagreements (65%) were with the ID physician. In the disagreements, a second ID physician agreed with IM residents in 12 of 20 cases (60%). The second ID physician agreed with the resident that the meropenem initiation was appropriate (when the initial ID physician or pharmacist deemed the initiation inappropriate) 11 of 12 times (91.7%) and that the meropenem was inappropriate (when the initial ID physician or pharmacist deemed the initiation appropriate) 1 of 12 times (8.3%). In all cases where the second ID physician agreed with the resident, they also agreed on the criteria. When examining IM resident agreement with either of the 2 ID physicians or ID pharmacist, it occurred in 89 of 97 (91.7%) cases (kappa 0.81, *P* <.001).

In the 8 cases where the second ID physician also disagreed with IM residents, the IM residents considered the initiation inappropriate in 5 of 8 cases (62.5%), while either of the two ID physicians or the ID pharmacist deemed it appropriate, agreeing on the same criteria. Conversely, in the remaining 3 of 8 cases (37.5%), the IM residents found the initiation appropriate based on criterion no. 4 as mentioned in Table 1 (Persistent fever or hemodynamic instability in a patient who is already receiving broad-spectrum β-lactam [agents with anti-pseudomonal coverage that are not carbapenems] for at least 48 hours), whereas either of the 2 ID physicians or the ID pharmacist considered it inappropriate.

The most common criterion for appropriate use of meropenem as determined by both IM residents and ID physician/pharmacist was initiation due to persistent fever or hemodynamic instability, despite having received broad-spectrum β-lactam treatment (agents with anti-pseudomonal coverage that are not carbapenems) for at least 48 hours (25.8% and 20.6%, respectively) (Table 3). Among the cases where IM residents and an ID physician/pharmacist agreed on appropriateness, they agreed on the same criterion for meropenem initiation 87% of the time, yielding a kappa statistic of 0.81 (*P* <.001).

**Table 3.** Criteria selected for appropriate empiric use of meropenem

Criteria	IM residents (number/%) [n = 55/97]	ID physician/pharmacist (number/%) [n = 47/97]
Severe infection and history of ESBL-infection/colonization or MDR *Pseudomonas aeruginosa* infection in the past 6 mo	10 (10.3%)	10 (10.3%)
Severe infection and at least 1 risk factor for ESBL-producing organisms in the past 6 mo	14 (14.4%)	14 (14.4%)
Severe infection and severe penicillin allergy	2 (2.1%)	3 (3.1%)
Persistent fever or hemodynamic instability in a patient who is already receiving broad-spectrum β-lactam (agents with anti-pseudomonal coverage that are not carbapenems) for at least 48 h	25 (25.8%)	20 (20.6%)
Nosocomial bacterial meningitis	1 (1.0%)	0 (0%)
Febrile neutropenia	3 (3.1%)	0 (0%)

Note. IM, internal medicine; ID, infectious diseases; ESBL, extended-spectrum beta-lactamase; MDR, multidrug resistant.

A comparison of participants (Table 4) based on meropenem administration appropriateness determined by ID physician/pharmacist showed that in patients where ID physician/pharmacist deemed appropriate meropenem use, the median days of meropenem use was 3 days (IQR 2, 6),

**Table 4.** Comparison of clinical characteristics based on meropenem administration appropriateness determined by ID clinician/pharmacist

Variable	Appropriate	Not appropriate	*P* value
LOS **(days/IQR)**	14 (8, 29)	14.5 (7, 28)	0.5536
Meropenem duration **(days/IQR)**	3.0 (2.0, 6.0)	2.0 (1.0, 5.0)	**0.045**
LOS after meropenem initiation **(days/IQR)**	3.0 (1.0, 10.0)	3.0 (1.0, 8.0)	0.852
ICU LOS **(days/IQR)**	5.2 (3.0, 11.9)	5.6 (2.4, 12.2)	0.994
ICU LOS after meropenem initiation **(days/IQR)**	4.0 (2.0, 6.0)	4 (2.0, 8.0)	0.968
In-hospital mortality **(number/%)**	19 (40.4%)	23 (46%)	0.580
ID consult **(number/%)**	44 (93.6%)	46 (92.0%)	>0.99
Admitting service **(number/%)**			0.084
Critical care (n = 77)	41 (53.3%)	36 (46.8%)	
Cardiology (n = 9)	3 (33.3%)	6 (66.7%)	
Surgery (n = 10)	2 (20%)	8 (80%)	
Trauma (n = 1)	1 (100%)	0 (0%)	

Note. LOS, length of stay; ICU, intensive care unit; ID, infectious diseases.

while those where empiric meropenem was deemed inappropriate have a median duration of 2 days (IQR 1, 5; *P* = 0.045). Other characteristics, including hospital and ICU LOS, in-hospital mortality, and admitting service, were not significantly different between the groups.

## Discussion

Meropenem is a widely used empiric antibiotic with bactericidal properties; it inhibits bacterial cell wall synthesis by binding to and inactivating penicillin-binding proteins.^28^ It has broad activity against gram-positive, gram-negative, and common anaerobic bacteria (eg, *Bacteroides fragilis*, *Bacteroides* group, and *Fusobacterium* spp.); however, meropenem has no coverage against *Enterococcus faecium*, methicillin-resistant *Staphylococcus aureus*, methicillin-resistant coagulase-negative *Staphylococci*, vancomycin-resistant *Enterococcus*, and *Stenotrophomonas maltophilia.* ^5,7^ It is FDA approved for the treatment of bacterial meningitis, complicated skin and skin structure infections, and bacterial meningitis. However, it can also be used for bloodstream infections, febrile neutropenia, bone and joint infections, complicated intra-abdominal infections, complicated UTI, obstetric and gynecological infections, severe community-acquired pneumonia, hospital-acquired pneumonia, and ventilator-associated pneumonia, if the infection is suspected to be caused by susceptible bacteria.^5^ Our study showed that meropenem was most used empirically to treat pneumonia (30.9%), similar to a previous study (31.7%).^13^

Antimicrobial stewardship has been defined as “the optimal selection, dosage, and duration of antimicrobial treatment that results in the best clinical outcome for the treatment or prevention of infection, with minimal toxicity to the patient and minimal impact on subsequent resistance.”^29^ However, there are no standardized definitions for appropriate and inappropriate therapy, and studies differ on the definition to assess the appropriateness of use. Most commonly, appropriate empiric therapy is defined as selecting an antimicrobial based on the suspected source of infection and disease severity.^29,30^ Another definition of appropriateness is the use based on practice guidelines, institution protocols, and accepted norms for the site of infection.^31^ We devised our own standards for meropenem appropriateness, drawing from past research guidelines^15,16^ and adapting them to suit our local protocols to reflect multidrug-resistant organism trends (Table 1).

Antimicrobial stewardship programs (ASP) help organizations ensure antibiotics are used appropriately by understanding local needs and prescriber behaviors. Key components like preauthorization, which requires clinician approval for certain antibiotics before prescription, and PAF, which engages providers post-prescription, effectively improve antibiotic use, reduce resistance, and lower *Clostridioides difficile* infection rates without increasing mortality.^32^ In our study, the general agreement between the primary ID physician/pharmacist and IM resident for appropriate administration of meropenem was in 79.4% of cases, with a kappa statistic indicating a weak agreement between the 2 parties.^33^ The agreement improved to 91.7% after a second ID physician reviewed the disagreements, indicating strong agreement. IM residents and an ID physician/pharmacist agreed on the same criterion for meropenem initiation 87% of the time, with kappa statistic indicating a strong agreement. There is a significant influence on the IM residents in the ICU with a large team of attendings and their individual preferences for antibiotics whereas during PAF residents are separated from the influence of others. This indicates that IM residents could contribute to ASP efforts using standardized criteria for appropriateness, as they share strong agreement with the “gold standard” ID physicians and pharmacists. This could be achieved by adding an ASP rotation during residency with independent reviews by IM residents using standardized criteria.

The duration of meropenem administration was shorter in patients where the ID physician/pharmacist deemed meropenem usage inappropriate. Also, inappropriate meropenem got discontinued earlier because culture data indicated no data for its use, or some patients had an ID consult and they deemed inappropriate use. Other characteristics were not different between the groups. These comparisons were not adjusted for potential confounders and may be under-powered as they were not the primary aim of the study, so they should be considered exploratory.

Our study confirms the high rates of inappropriate use of meropenem in critically ill patients, indicating it is an important area of focus for ASP efforts. To our knowledge, it is the first study to use IM residents as evaluators of appropriateness. Our study has limitations. It was performed at a single center, a teaching community hospital ICU, so results may not be generalizable to other types of hospitals. The sample size was small, but we were able to identify that approximately half of the meropenem orders were inappropriate and kappa statistic *P* values were significant. This was a retrospective study, but we minimized selection bias by including all patients who had meropenem initiated in the ICU during the study period regardless of any other baseline characteristics. Appropriateness was determined based on chart review which relied on documentation of the patient’s history, course, and comorbidities, which may not always be complete or accurate. Indeed, the IM residents missed or overcalled some criteria (allergy status, nosocomial bacterial meningitis, febrile neutropenia, and persistent fever/hemodynamic instability) as shown in Table 3, indicating that careful chart review is necessary and may require prior training, especially if residents will be used in the PAF process which relies on meticulous chart review.

This study highlights a significant prevalence of improper empiric meropenem usage among critically ill patients. Nevertheless, it also demonstrates that IM residents can aid in antimicrobial stewardship initiatives by employing standardized criteria to determine appropriateness.

## References

[1] Dellinger RP , Levy MM , Carlet JM , et al. Surviving Sepsis Campaign: international guidelines for management of severe sepsis and septic shock. Crit Care Med 2008;36:296–327. 18158437 10.1097/01.CCM.0000298158.12101.41

[2] Leekha S , Terrell CL , Edson RS. General principles of antimicrobial therapy. Mayo Clin Proc 2011;86:156–167. 21282489 10.4065/mcp.2010.0639 PMC3031442

[3] Zilberberg MD , Shorr AF , Micek ST , Vazquez-Guillamet C , Kollef MH. Multi-drug resistance, inappropriate initial antibiotic therapy and mortality in Gram-negative severe sepsis and septic shock: a retrospective cohort study. Crit Care 2014;18:596. 25412897 10.1186/s13054-014-0596-8 PMC4264255

[4] Dadgostar P. Antimicrobial resistance: implications and costs. Infect Drug Resist 2019;12:3903–3910. 31908502 10.2147/IDR.S234610 PMC6929930

[5] Baldwin, C.M. , Lyseng-Williamson, K.A. & Keam, S.J. Meropenem. Drugs 2008:68:803–838. 18416587 10.2165/00003495-200868060-00006

[6] Temkin E , Fallach N , Almagor J , et al. Estimating the number of infections caused by antibiotic-resistant *Escherichia coli* and *Klebsiella pneumoniae* in 2014: a modelling study. Lancet Glob Health 2018;6:e969–e979. 30103998 10.1016/S2214-109X(18)30278-X

[7] Duffy J , Sievert D , Rebmann C , et al. Effective state-based surveillance for multidrug-resistant organisms related to health care-associated infections. Public Health Rep 2011;126:176–185. 21387947 10.1177/003335491112600208 PMC3056030

[8] World Health Organization. WHO Bacterial Priority Pathogens List, 2024: Bacterial Pathogens of Public Health Importance to Guide Research, Development and Strategies to Prevent and Control Antimicrobial Resistance. World Health Organization, 2024. https://www.who.int/publications/i/item/9789240093461.

[9] Salehifar E , Shiva A , Moshayedi M , Kashi TS , Chabra A. Drug use evaluation of Meropenem at a tertiary care university hospital: A report from Northern Iran. J Res Pharm Pract 2015;4:222–225. 26645030 10.4103/2279-042X.167047 PMC4645136

[10] Tarcea Bizo P , Dumitras D , Popa A. Evaluation of restricted antibiotic use in a hospital in Romania. Int J Clin Pharm 2015;37:452–456. 25832678 10.1007/s11096-015-0096-1

[11] Khan MU , Yousuf RI , Shoaib MH. Drug utilization evaluation of meropenem and correlation of side effects with renal status of patients in a teaching based hospital. Pak J Pharm Sci 2014;27:1503–1508. 25176244

[12] Raveh D , Muallem-Zilcha E , Greenberg A , Wiener-Well Y , Schlesinger Y , Yinnon AM. Prospective drug utilization evaluation of three broad-spectrum antimicrobials: cefepime, piperacillin-tazobactam and meropenem. QJM 2006;99:397–406. 16682440 10.1093/qjmed/hcl050

[13] Naderi P , Shirani K , Soltani R , Khorvash F , Naji Esfahani SS. Meropenem Utilization Evaluation in a Referral Teaching Hospital in Iran. J Res Pharm Pract 2018;7:83–87. 30050961 10.4103/jrpp.JRPP_17_86 PMC6036873

[14] Alba Fernandez J , Del Pozo JL , Leiva J , et al. Impact of the acceptance of the recommendations made by a meropenem stewardship program in a university hospital: a pilot study. Antibiotics (Basel) 2022;11:330. 35326793 10.3390/antibiotics11030330 PMC8944864

[15] Al-Hadithi D , Al-Zakwani I , Balkhair A , Al Suleimani YM. Evaluation of the appropriateness of meropenem prescribing at a tertiary care hospital: a retrospective study in Oman. Int J Infect Dis 2020;96:180–186. 32339716 10.1016/j.ijid.2020.04.045

[16] Janssen J , Kinkade A , Man D. CARBapenem utilization evaluation in a large community hospital (CARBON): a quality improvement study. Can J Hosp Pharm 2015;68:327–331. 26327707 10.4212/cjhp.v68i4.1473 PMC4552234

[17] Macy E , Ngor E. Recommendations for the management of beta-lactam intolerance. Clin Rev Allergy Immunol 2014;47:46–55. 23549754 10.1007/s12016-013-8369-8

[18] Lee Y , Bradley N. Overview and insights into carbapenem allergy. Pharmacy (Basel) 2019;7:110. 31398843 10.3390/pharmacy7030110 PMC6789495

[19] Picard M , Robitaille G , Karam F , et al. Cross-reactivity to cephalosporins and carbapenems in penicillin-allergic patients: two systematic reviews and meta-analyses. J Allergy Clin Immunol Pract 2019;7:2722–2738.e5. 31170539 10.1016/j.jaip.2019.05.038

[20] Ben-Ami R , Rodríguez-Baño J , Arslan H , et al. A multinational survey of risk factors for infection with extended-spectrum beta-lactamase-producing Enterobacteriaceae in nonhospitalized patients. Clin Infect Dis 2009;49:682–690. 19622043 10.1086/604713

[21] Paterson DL , Ko WC , Von Gottberg A , et al. International prospective study of *Klebsiella pneumoniae* bacteremia: implications of extended-spectrum beta-lactamase production in nosocomial Infections. Ann Intern Med 2004;140:26–32. 14706969 10.7326/0003-4819-140-1-200401060-00008

[22] Park YS , Adams-Haduch JM , Shutt KA , et al. Clinical and microbiologic characteristics of cephalosporin-resistant Escherichia coli at three centers in the United States. Antimicrob Agents Chemother 2012;56:1870–1876. 22290945 10.1128/AAC.05650-11 PMC3318325

[23] Kang CI , Wi YM , Lee MY , et al. Epidemiology and risk factors of community onset infections caused by extended-spectrum β-lactamase-producing Escherichia coli strains. J Clin Microbiol 2012;50:312–317. 22162561 10.1128/JCM.06002-11 PMC3264158

[24] Rodríguez-Baño J , Picón E , Gijón P , et al. Community-onset bacteremia due to extended-spectrum beta-lactamase-producing Escherichia coli: risk factors and prognosis. Clin Infect Dis 2010;50:40–48. 19995215 10.1086/649537

[25] Lee JA , Kang CI , Joo EJ , et al. Epidemiology and clinical features of community-onset bacteremia caused by extended-spectrum β-lactamase-producing *Klebsiella pneumoniae* . Microb Drug Resist 2011;17:267–273. 21388296 10.1089/mdr.2010.0134

[26] Nakai H , Hagihara M , Kato H , et al. Prevalence and risk factors of infections caused by extended-spectrum β-lactamase (ESBL)-producing Enterobacteriaceae. J Infect Chemother 2016;22:319–326. 26968486 10.1016/j.jiac.2016.02.004

[27] Briongos-Figuero LS , Gómez-Traveso T , Bachiller-Luque P , et al. Epidemiology, risk factors and comorbidity for urinary tract infections caused by extended-spectrum beta-lactamase (ESBL)-producing enterobacteria. Int J Clin Pract 2012;66:891–896. 22897466 10.1111/j.1742-1241.2012.02991.x

[28] Bax RP , Bastain W , Featherstone A , et al. The pharmacokinetics of meropenem in volunteers. *J Antimicrob Chemother* 1989;24:311–320. 10.1093/jac/24.suppl_a.311 2808215

[29] Kollef MH , Sherman G , Ward S , Fraser VJ. Inadequate antimicrobial treatment of infections: a risk factor for hospital mortality among critically ill patients. Chest 1999;115:462–474. 10027448 10.1378/chest.115.2.462

[30] Kumar A , Ellis P , Arabi Y , et al. Initiation of inappropriate antimicrobial therapy results in a fivefold reduction of survival in human septic shock. Chest 2009;136:1237–1248. 19696123 10.1378/chest.09-0087

[31] DePestel DD , Eiland EH , Lusardi K , Destache CJ , Mercier R-C , McDaneld PM , Lamp KC , Chung TJ , Hermsen ED . Assessing appropriateness of antimicrobial therapy: in the eye of the interpreter. Clin Infect Dis 2014; 59:S154–S161. 25261542 10.1093/cid/ciu548

[32] Elligsen M , Walker SA , Pinto R , et al. Audit and feedback to reduce broad-spectrum antibiotic use among intensive care unit patients: a controlled interrupted time series analysis. Infect Control Hosp Epidemiol 2012;33:354–361. 22418630 10.1086/664757

[33] McHugh ML. Interrater reliability: the kappa statistic. Biochem Med (Zagreb) 2012;22:276–282. 23092060 PMC3900052

